# Supplementary material for: Content-rich biological network constructed by mining PubMed abstracts
Source: BMC Bioinformatics. 2004 Oct 8;5:147. doi: 10.1186/1471-2105-5-147 (PMC528731; doi:10.1186/1471-2105-5-147)
Supplement: Additional File 2 — The original results of the above study (non-essential files are deleted to keep the file size under the limit set by BMC bioinformatics). [file 1471-2105-5-147-S2.bz2 › chilibotAdditionalFile2/dip10/761ID8917517E18148/html/DNAB_DNAG.html]

 


 **DNAB** and **DNAG** 
  
Found 91 abstracts in PubMed, retrieved 10.  
 

 What does Google say? 
 PDF only 
| .edu only 

---

**Interactive relationship** (e.g. stimulation, inhibition, etc)

**Neutral relationship**- Mapping protein protein interactions within a stable complex of DNA primase  [ **DNAG** ]  and  **DnaB**  helicase from Bacillus stearothermophilus.  Ref: 10625492 Biochemistry, 2000
- Identification of a region of Escherichia coli  **DnaB**  required for functional interaction with  **DnaG**  at the replication fork.  Ref: 10833513 J Biol Chem, 2000
- Mutations in this region of  **DnaB**  affect the  **DnaB**   **DnaG**  interaction during both general priming directed and phiX174 complementary strand DNA synthesis, as well as at replication forks reconstituted in rolling circle DNA replication reactions.  Ref: 10833513 J Biol Chem, 2000
- As we have demonstrated previously,  **DnaG**  is recruited to the replication fork via a transient protein protein interaction with  **DnaB** .  Ref: 10833513 J Biol Chem, 2000
- ... seven proteins, PriA, PriB, PriC, DnaT,  **DnaB** , DnaC, and  **DnaG** , were required for primosome assembly on phi X174 DNA, only  **DnaB** , DnaC, and  **DnaG**  were required for replication from oriC, suggesting that the other proteins were not involved in chromosomal replication.  Ref: 10506828 Prog Nucleic Acid Res Mol Biol, 1999
- Homogenous assays for Escherichia coli  **DnaB**  stimulated  **DnaG**  primase and  **DnaB**  helicase and their use in screening for chemical inhibitors.  Ref: 12009693 Anal Biochem, 2002

**Non-interactive relationship** (e.g. studied together, co-existance, homology, etc.)

- This can be attributed to its ability, unlike wild type DnaC, to catalyze a SSB insensitive general priming reaction with  **DnaB**  and  **DnaG**  on any SSB coated single stranded DNA.  Ref: 10713144 J Biol Chem, 2000
- In Escherichia coli, the primosome assembly proteins, PriA, PriB, PriC, DnaT, DnaC,  **DnaB** , and  **DnaG** , are thought to help to restart DNA replication forks at recombinational intermediates.  Ref: 10835375 Genetics, 2000
- The  **DnaB**  homolog from Arabidopsis also contains a  **DnaG**  primase domain and the  **DnaB**  homolog from the nematode seems to contain an inactivated version of the primase.  Ref: 10645945 Genome Res, 2000
- The behavior of the purified mutant  **DnaB**  proteins in the various replication systems suggests that access to the  **DnaG**  binding pocket on  **DnaB**  may be restricted at the replication fork.  Ref: 10833513 J Biol Chem, 2000
- However, recombination was stimulated by mutations in genes encoding replication proteins, including the primosomal proteins  **DnaB** , DnaD and the  **DnaG**  primase, the putative DNA polymerase III subunits PolC, DnaN and DnaX, as well as the DNA polymerase DnaE.  Ref: 11251841 Mol Microbiol, 2001
- Using a combination of biochemical and physical techniques, we show that the helicase primase complex comprises one  **DnaB**  hexamer and either two or three molecules of  **DnaG** .  Ref: 10625492 Biochemistry, 2000
- For the first time, we demonstrate directly a stable complex between a bacterial  **DnaG**  primase and  **DnaB**  helicase.  Ref: 10625492 Biochemistry, 2000
- The replication restart proteins, PriA, PriB, PriC,  **DnaB** , DnaC,  **DnaG** , DnaT and Rep, form redundant pathways that recognize repaired replication forks and restart them.  Ref: 11532137 Mol Microbiol, 2001
- The fundamental activities of the replicative primosomes of Escherichia coli are provided by  **DnaB** , the replication fork DNA helicase, and  **DnaG** , the Okazaki fragment primase.  Ref: 10833513 J Biol Chem, 2000
- The assay uses an adaptation of the general priming reaction by employing  **DnaG**  primase,  **DnaB**  helicase, and ribonucleotidetriphosphates incorporation of 3 H CTP for in vitro primer synthesis on single stranded oligonucleotide and M13mp18 DNA templates.  Ref: 12009693 Anal Biochem, 2002
